# Attitudes towards conscientious objection among community pharmacists in Poland

**DOI:** 10.1007/s11096-013-9878-5

**Published:** 2013-11-15

**Authors:** Anna Piecuch, Malwina Gryka, Małgorzata Kozłowska-Wojciechowska

**Affiliations:** Department of Clinical Pharmacy and Pharmaceutical Care, Medical University of Warsaw, ul. Banacha 1, PL02-097 Warsaw, Poland

**Keywords:** Conscience clauses, Pharmacy, Poland, Professional ethics, Refusal clauses, Religion

## Abstract

*Background* The European Council Resolution 1763 (2010), “The right to conscientious objection in lawful medical care”, provoked a discussion among Polish pharmacists on the necessity for granting them the right to refuse to dispense medicinal products which invoke conscientious objection. *Objective* To explore attitudes of Polish pharmacists towards the conscience clause. *Setting* Pharmacies with public e-mail addresses in various parts of Poland (Lower Silesia Province, Mazovia Province, Kuyavia-Pomerania Province, and West Pomeranian Province). *Method* An online survey questionnaire addressed to 1,454 pharmacies. The participants were asked 8 questions, including a question addressed only to pharmacy managers and owners. *Main outcome measure* Attitudes towards the right to conscientious objection for pharmacists. *Results* Ultimately, responses of 126 pharmacists (83 women, 43 men, average age-39 years of age) were taken into consideration. Most participants (92 %) have never refused to fill a prescription due to their beliefs; however, 15 % of participants state that if the conscience clause were legally sanctioned, they would exercise this right. Most participants (73 %) think that pharmacists should not have the right to conscientious objection. Almost half of participants who support implementation of the conscience clause would grant this right to pharmacists on a conditional basis, if the pharmacists were obliged to present other real options to the patient about obtaining a specific product. *Conclusion* Pharmacists are rather reluctant to the idea of implementing the conscience clause, but despite a clear majority of its opponents, there seems to be a necessity for introducing such a regulation.

## Impact of findings on practice

- A situation when professional pharmacy employees refuse to dispense a medication on non-legally sanctioned grounds requires urgent regulation.
- Pharmacists’ role in patient care exceeds technical acts, and therefore pharmacists should have the professional right to deal with ethical dilemmas according to their conscience.
- The Polish Pharmaceutical Chamber should draw up standards for professional behavior to guide the pharmacists’ decision making when ethical dilemmas arise in practice.
- Professional education in pharmacy ethics should put more emphasis on dealing with ethical dilemmas in professional practice.

## Introduction

In Poland, the right to conscientious objection, commonly referred to as the conscience clause, is granted to physicians [1], nurses and obstetric nurses [2]. It is also a subject of some pharmacists’ claims. An argument that strengthens the pharmacists’ pursuit of implementation of this solution is the European Council Resolution 1763 (2010), *The right to conscientious objection in lawful medical care*, which appeals to respect the right to freedom of conscience among persons who do not want to participate in the procedures of abortion or euthanasia as well as the activities that may lead to the death of a human embryo or foetus [3]. According to the Resolution, persons who refuse to participate in these procedures shall not be coerced, discriminated or held liable because of their decision. However, the adopted legal solutions should also ensure that each patient is able to access medical care.

In Poland, the conscientious objection of pharmacists is mainly discussed with regard to hormonal contraception, including emergency contraceptives, and intrauterine devices [4] as well as aborted human foetal-cell line vaccines. Each of these products requires a medical prescription to be filled only in pharmacies and dispensaries [5]. Due to a limited access to medicinal products on prescription, Polish legal acts provide a clear definition of situations when a pharmacist has the right and even obligation to refuse to dispense a medicinal product [6, 7]. Among applicable regulations, there is no regulation which gives an opportunity to refuse a pharmacy service which violates a pharmacist’s conscience.

The priority values and general standards of ethical conduct to be observed by Polish pharmacists are set out in the Code of Ethics for Pharmacists of the Republic of Poland [8]. The Code of Ethics guarantees pharmacists, as persons accountable for work they perform, freedom to act according to their conscience and current medical knowledge. Under the Code of Ethics previously in force, pharmacists whose beliefs prevented them from performing particular services were required to refer patients to another pharmacist for the service they required [9]. In some other countries, this principle gives pharmacists the option to refuse to dispense a particular medicinal product on the grounds of their beliefs, while at the same time ensuring that the patient has access to the medicine [10, 11]. This provision has been omitted from the current Code. A pharmacist in Poland is currently unable to refuse to fill a prescription for a medicinal product on moral, ethical or religious grounds. The European Council Resolution 1763 (2010) on conscientious objection is not binding until it has been implemented in national law. At the same time, Poland is not required to implement this provision. The Code of Ethics for Pharmacists of the Republic of Poland is a typical corporate code, which is also not legally binding. Therefore, in practice pharmacists are not in a position to refuse to dispense a medicinal product due to conscientious objection. Moreover, Polish Pharmaceutical Chamber has yet to take an official position on pharmacists’ conscientious objection.

## Aim of the study

The study is designed to explore attitudes of Polish pharmacists towards a so-called conscience clause, i.e. the right to refuse to dispense a medicinal product which invokes the conscientious objection of a pharmacist.

## Methods

The study was conducted over a period of 8–28 May 2012. The study research tool was a survey questionnaire, designed on the basis of the research team’s discussions and literature review. An anonymous questionnaire, addressed to pharmacists and pharmacy owners, was emailed to 1,454 pharmacies with public e-mail addresses among all 2,725 pharmacies in the region (515 pharmacies in Lower Silesia Province, 848 pharmacies in Mazovia Province, 45 pharmacies in Kuyavia and Pomerania Province as well as 46 pharmacies in West Pomeranian Province). The addresses of pharmacies were acquired through the Provincial Pharmaceutical Inspectorates in Warsaw, Bydgoszcz, Szczecin and Wrocław. With regard to questionnaire completion, there was no time limitation. Respondents’ answers were collected in a virtual file on an ongoing basis.

The questionnaire consisted of two parts. In the first part, seven closed-ended question items on the respondents’ opinions towards conscientious objection as well as one closed-ended question item, addressed only to pharmacy managers and owners, were included. The other part of the questionnaire contained demographic characteristics. The statistical analysis was carried out using STATISTICA 9.1 software.

Ethical approval was not required for this study.

## Results

Due to invalid or wrong email addresses, 40 out of 1,454 pharmacies did not receive a message. During the first week, 121 filled questionnaires were provided. Due to a low response rate, the second request for participation was sent to the pharmacies and, during the next 2 weeks, additional 12 filled questionnaires were obtained. Eventually, 133 respondents participated in the study (the response rate was 9.4 % of 1,414 delivered emails). The highest response level was recorded during the first 2 days of the study.

Among the 133 responses, one incomplete questionnaire and 6 questionnaires filled by non-pharmacists were excluded from the research. Ultimately, responses of 126 participants, aged 25–65 (average age—39 years of age) were taken into consideration. Due to a specific nature of the subject, the participants were asked about their religion. Three quarters (95 participants) stated they belong to the Catholic Church, while 13 % (16 participants) refused to answer. The other participants reported a non-Catholic religion (3) or no religion (12). The religion data seem to be consistent with official Polish statistics. According to 2008 data from the Polish Central Statistical Office (GUS), around 88.4 % of Polish citizens are Catholics, while 9.3 % declare no religion [12]. In Table 1, the socio demographic characteristics of the participants are presented.

**Table 1:** The socio demographic characteristics of the study group

Independent variables	*n* = 126
Age (years)	39 ± 10
Gender	
Women	83 (66 %)
Men	43 (34 %)
Job experience (years)	
≤5	29 (23 %)
6–10	24 (19 %)
11–15	31 (25 %)
26–20	25 (20 %)
>20	17 (13 %)
Position^a^	
Pharmacist (Master of Sciences in Pharmacy)	126 (100 %)
Pharmacy manager	61 (48 %)
Pharmacy owner	38 (30 %)
Pharmacy location	
Village	17 (13 %)
Town (a population of up to 20,000)	17 (13 %)
Town (a population of more than 20,000–100,000)	39 (31 %)
City (a population of more than 100,000–500,000)	8 (6 %)
City (a population of more than 500,000)	45 (36 %)
Pharmacy	
Independent	94 (75 %)
Chain	32 (25 %)

^a ^The answers do not add up to 100 % as the participants could select more than one answer

Most participants (92 %) stated they had never refused to fill a prescription due to their beliefs. Only 8 % of participants admitted they had not filled a prescription due to their beliefs. Most participants (67 %) state that even if the conscience clause were legally sanctioned, they would not exercise this right, while 15 % of participants would exercise the right to conscientious objection, if it were required by legislation. The other 17 % of participants could not state whether or not they would make use of this right.

Most participants (74 %) think that pharmacists should not have the right to conscientious objection. Among 31 participants who supported implementation of the conscience clause, 14 participants would grant this right to pharmacists on the conditional basis, if the pharmacists who refused to dispense a product due to their beliefs were obliged to present other real options to the patient about obtaining a given product. Two participants could not provide their opinions on the issue.

The participants (25 %) who declared their support for the right to conscientious objection for pharmacists were asked to explain the reasons affecting their attitudes to the conscience clause. Most of those participants (84 %) support the conscience clause for pharmacists because, in their opinion, pharmacists should be granted the right to act according to their conscience. The participants’ answers are listed in Table 2.

**Table 2:** The reasons for supporting the conscientious objection in the pharmacy profession (*n* = 31)^a^

The reason for supporting conscientious objection	Participants (%)
Moral beliefs	12 (39)
Religious norms	10 (8)
Cultural norms	7 (6)
A pharmacist should have the right to act according to his/her conscience	26 (84)

^a ^The answers do not add up to 100 % as the participants could select more than one answer

The opponents (93 participants) of legally sanctioned conscientious objection in the pharmacy profession were asked to explain the reasons of their opinion. Most of them (78 %) think that a pharmacist must not impose his/her own views and beliefs on patients. Slightly fewer participants (70 %) emphasise that patients should not have problems with the access to medicinal products they are legally entitled to and which are only available in pharmacies. In Table 3, the answers of the opponents of the conscience clause for pharmacists are presented.

**Table 3:** The reasons for opposing the conscience clause (*n* = 93)^a^

The reason for opposing the conscience clause	*n* = 93 (100 %)
A pharmacist should always give the first priority to a patient’s interest	49 (53 %)
A pharmacist does not have sufficient knowledge about medical indications as the basis for prescribing a specific product	42 (45 %)
Patients should not have problems with the access to medicinal products they are legally entitled to which are only available in pharmacies	71 (76 %)
A pharmacist must not impose his/her own views and beliefs on patients	82 (88 %)
Other	2 (2 %)

^a^The answers do not add up to 100 % as the participants could select more than one answer

Among 126 participants, 80 participants (63 %) think that if the right to conscientious objection for pharmacists were legally sanctioned, it should be limited to a specific list of products. In the opinion of the other participants, there is no need of such a restriction.

The participants were asked about the products which should be subject to the conscience clause, if the right to conscientious objection were limited to specific agents. The suggested answers were selected based on the available worldwide publications regarding pharmacists’ conscientious objection and press releases related to the discussion on the conscience clause for pharmacists in Poland. The question was answered by 68 participants. Table 4 presents a list of products which, in the participants’ opinion, should be subject to the conscience clause.

**Table 4:** Products which should be subject to the conscience clause (*n* = 80)^a^

Products which should be subject to the conscience clause	Participants, *n* = 80 (%)
Emergency contraception	52 (65)
Hormonal contraception	17 (21)
Intrauterine devices	22 (27)
Other forms of contraception	11 (14)
Anti-erectile dysfunction agents	5 (6)
Vaccines	37 (46)
Other	2 (3)

^a^ The answers do not add up to 100 % as the participants could select more than one answer

In the question addressed only to pharmacy managers and owners (77), the participants were asked if they would allow their employees to exercise the right to conscientious objection if it were legally sanctioned. 31 participants would allow their employees to make use of the legally sanctioned right to conscientious objection, while 29 participants would refuse. 18 participants could not provide a clear opinion on the issue.

## Discussion

We decided to conduct the research with the use of an online survey questionnaire because of the peculiarity and cultural sensitivity of the topic. However, the online questionnaires were only received by the pharmacies with e-mail addresses available in the registers of pharmacies kept by the Provincial Pharmaceutical Inspectorates. The surveyed pharmacies represented over 50 % of all pharmacies located in the participating provinces.

In 2011, generally accessible pharmacies and pharmacy outlets in Poland employed a total of 24.3 thousand pharmacists (83.9 % women)[13]. The higher rate of men (43 %) among the research participants may be related to a higher rate of male pharmacy managers and owners.

According to most pharmacists, there is no necessity for granting pharmacists the right to conscientious objection, but a quarter of participants find it necessary and this rate is high enough not to be neglected.

Almost half of the proponents of the conscience clause among the participants would grant this right provided that a pharmacist were obliged to present real possible options to the patient about obtaining the product from another pharmacist or pharmacy. This seems to be an option that balances arguments in favour and against a pharmacist right to object [14]. An analogical, legally sanctioned conditional right to conscientious objection is granted to physicians and nurses. If such a regulation were implemented, pharmacists would be allowed to refuse to dispense a product which invokes their conscientious objection on the one hand, while on the other hand, patients and their right to buy medicinal products they were entitled to would be protected.

Three quarters of the opponents of the conscience clause think that patients’ access to medicinal products they are legally entitled to and which are only available in pharmacies should not be hampered by pharmacists. This opinion is consistent with the decision of the European Court of Human Rights, regarding the case of Pichon and Sajous versus France, according to which pharmacists may not reject valid medical prescriptions and thus prevent patients from the access to medicinal products [15]. Moreover, the Court emphasised that pharmacists should not allow their personal convictions and beliefs to be superior to their professional responsibilities and goals. More than half of opponents of the conscience clause thought a pharmacist should always give the first priority to a patient’s interest even if it is in conflict with his/her own beliefs. This opinion is consistent with the Code of Ethics for Pharmacists of the Republic of Poland [8].

Most proponents of conscientious objection think a pharmacist should have the right to act according to his/her conscience. The freedom of conscience in Poland is guaranteed by the Constitution of the Republic of Poland [16]. However, regarding conscientious objection, we should consider which of the rights is superior: the pharmacists’ right to freedom of conscience or the patients’ right to obtain the service they are entitled to.

In the opinion of most participants, if the right to conscientious objection were implemented, it should be limited to a specific list of products. The most commonly selected agents were various types of contraception. A debate on the conscience clause is typically related to emergency contraception and this form was most frequently selected by the participants as the product subject to the conscience clause. An American study showed that emergency contraceptives, following medical abortion, were also indicated as the most common products that might invoke a pharmacist’s conscientious objection [17].

The second most frequently selected answer referred to aborted human foetal cell line vaccines. The cell lines that are currently used for production of certain types of vaccines were obtained in the 1960s from an aborted human foetus [18]. The ethical dilemma related to those vaccines refers to the concern whether rescuing lives and health of many people is a higher priority than the life of a foetus whose cells served as the material for vaccine production.

Only 8 % of the participants admitted they had refused to fill a prescription due to their beliefs, while in the opinion of 15 %, they would exercise the right to conscientious objection if it were legally sanctioned. This difference may imply that about 7 % of the participants sometimes dispense medications against their consciences. A relatively high rate (17 %) of participants who could not clearly state whether they would apply the conscience clause in their job if it were legally sanctioned may be explained in terms of situation-specific reaction. Those participants may have never experienced a direct conflict of conscience but they do not exclude it in the future.

Polish pharmacists should be guided in their work by expertise, human values, pharmaceutical traditions and applicable laws [8]. In the present circumstances, the role of pharmacists seems to be reduced to technical procedures performed within applicable laws, which leaves them no room to exercise their own judgement. An exception allowing pharmacists to act according to their conscience would raise their professional status, putting them on a par with doctors and nurses. Pharmacists duties to patients can be balanced with personal beliefs and conscientious objections through professional ethical deliberation.

The pharmaceutical community has little impact on applicable laws, which depend on the current political context and are created first and foremost by persons outside of the pharmaceutical profession. Nevertheless, Polish Pharmaceutical Chamber could put forward principles to provide directions and guidance to pharmacists who are faced with ethical dilemmas in everyday practice, enabling them to reconcile their own beliefs with the rights and needs of the patient without compromising professional values.

It seems that ethical education (in the framework of both undergraduate education and lifelong learning) could help pharmacists deal with ethical dilemmas faced in everyday practice. This approach could be studied further.

## Conclusion

On the one hand, a pharmacist is obliged to obey the pharmaceutical law, while on the other hand, ethical codes highlight the roles of conscience and the priority of professional ethics. Every day, some of pharmacy employees confront a dilemma whether they should satisfy patient’s expectations or act according to their conscience. Patient’s expectations not always meet moral beliefs of a pharmacist.

The study findings show that despite a clear majority of the opponents of conscientious objection in Poland, there is a large group of professional pharmacy employees who find implementation of such a regulation necessary. This opinion is confirmed by the fact that some of the participants admitted they had refused to fill a prescription for a medication that invoked their conscientious objection. A situation when professional pharmacy employees refuse to dispense a medication on non-legally sanctioned grounds is unacceptable and requires urgent regulation. Even more participants declare to exercise this right if it were legally sanctioned. Currently, a range of products that invoke conscientious objection of some Polish pharmacists is limited mostly to birth control medications. The problem may become worse in the future if more controversial products, e.g. for abortion or euthanasia, are placed on the market in Poland. Therefore, it also seems that more emphasis should be put on education in pharmacy ethics as new ethical dilemmas arise.
